# STIMulating blood pressure

**DOI:** 10.7554/eLife.77978

**Published:** 2022-03-24

**Authors:** Tessa AC Garrud, Jonathan H Jaggar

**Affiliations:** 1 Department of Physiology, University of Tennessee Health Science Center Memphis United States

**Keywords:** Stromal Interaction Molecules, store-operated calcium entry, blood pressure, peripheral coupling sites, STIM1, smooth muscle, Mouse

## Abstract

The protein STIM1 helps to maintain membrane coupling sites in smooth muscle cells that regulate arterial contractility and blood pressure.

**Related research article** Krishnan V, Ali S, Gonzales AL, Thakore P, Griffin CS, Yamasaki E, Alvarado MG, Johnson MT, Trebak M, Earley S. 2022. STIM1-dependent peripheral coupling governs the contractility of vascular smooth muscle cells. *eLife* **11**:e70278. doi: 10.7554/eLife.70278.

Arteries contain smooth muscle cells, which contract and relax to change the diameter of blood vessels, controlling blood flow to ensure that cells get the correct amount of oxygen and nutrients. To understand how blood flow and blood pressure are regulated, it is important to characterize signaling mechanisms that occur in these cells and change how they contract and relax. This information can then be used to determine what happens to these signaling pathways during cardiovascular diseases, such as hypertension and stroke, and develop new therapies to treat these life-threatening conditions.

In many cell types, including arterial smooth muscle cells, the plasma membrane surrounding the cell lies only a few nanometers away from the membrane of an organelle known as the endoplasmic reticulum, or in the case of muscle cells, the sarcoplasmic reticulum (SR/ER). The tiny cytoplasmic regions between these two membranes, called peripheral coupling sites, have their own microenvironment, where local communication can occur between proteins without impacting the rest of the cell. In muscle cells, these regions are involved in calcium signaling, which is responsible for regulating both contraction and relaxation (Figure 1). Despite the importance of peripheral coupling sites, how they form remains unclear.

**Figure 1.**
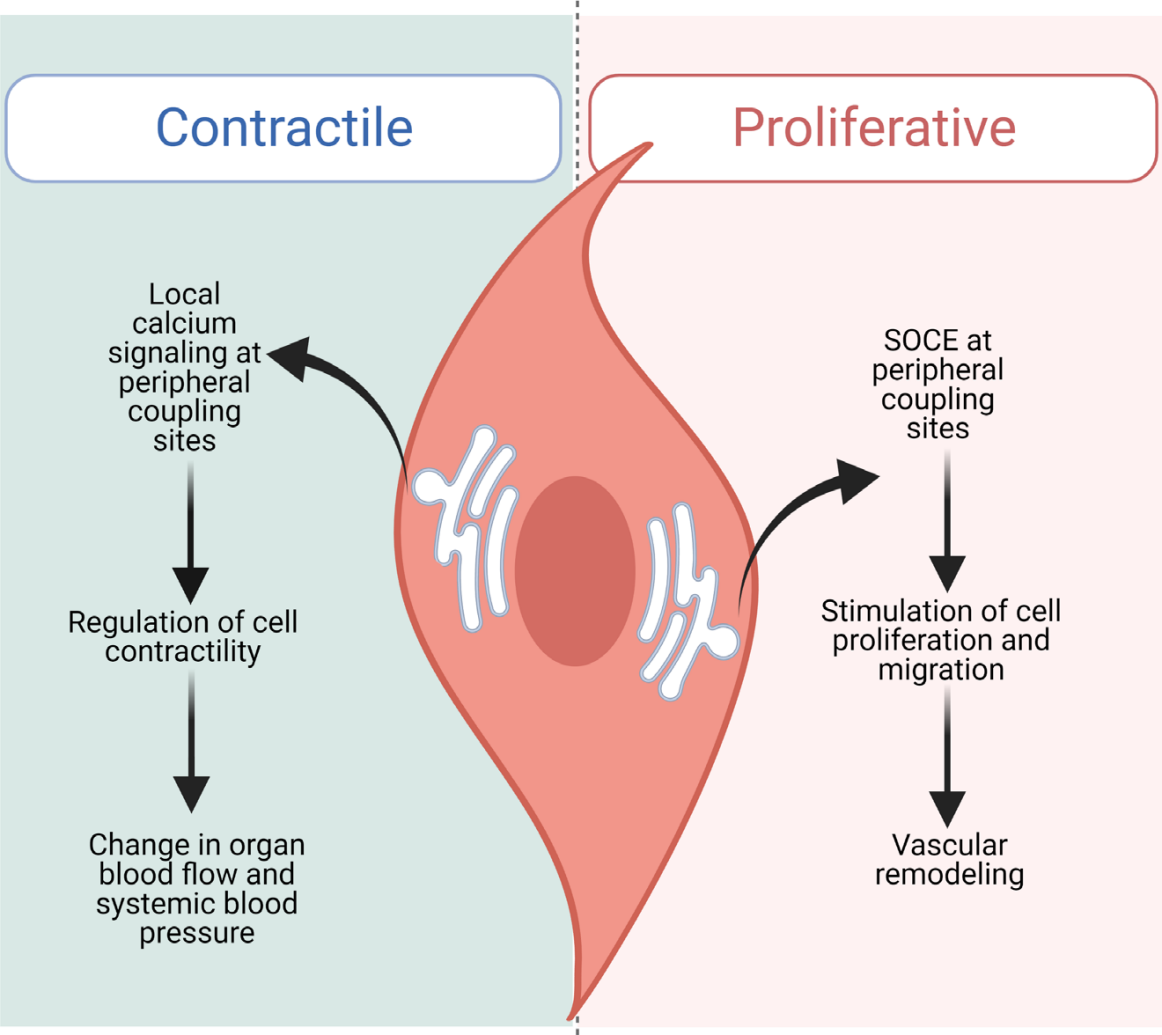
STIM1 performs distinct functions in contractile and proliferative arterial smooth muscle cells. In contractile smooth muscle cells (left), local increases in intracellular calcium concentration occur at peripheral coupling sites, allowing signaling to take place between proteins located in the SR/ER membrane and the plasma membrane without interfering with the rest of the cell. This signaling mechanism regulates smooth muscle cells within the walls of arteries, which contract and relax to modulate how much blood can flow to organs, controlling systemic blood pressure. Krishnan et al. found that loss of STIM1 reduces peripheral coupling sites in contractile arterial smooth muscle cells. This affects local signaling mechanisms, leading to over-dilated vessels and a decrease in blood pressure. Injury or disease of the vasculature (right) can shift arterial smooth muscle cells into a non-contractile, proliferative and migratory state. In this state, STIM1 does not maintain peripheral coupling sites, and instead takes on its canonical role inducing store-operated calcium entry (SOCE), which stimulates cells to multiply and migrate, altering the structure of blood vessels.

Image credit: Figure created using BioRender.

In many cell types, the membrane of the SR/ER contains proteins called Stromal Interaction Molecules (STIMs). These proteins are well known for playing a role in store operated calcium entry (SOCE), a mechanism that replenishes the calcium in the SR/ER. When the calcium concentration inside the SR/ER decreases, STIMs elongate and interact with ion channels on the plasma membrane termed Orai. These channels have many roles, including helping refill the SR/ER with calcium (Prakriya and Lewis, 2015).

This canonical STIM signaling pathway, however, does not occur in healthy arterial smooth muscle cells, which – despite having STIMs – have very little Orai and generate little to no SOCE (Krishnan et al., 2022; Potier et al., 2009; Xi et al., 2008). This apparent paradox has raised a decade-old question: what is the function of STIMs in arterial smooth muscle cells? Now, in eLife, Scott Earley and colleagues at the University of Nevada, the University of Pittsburgh and Pennsylvania State University – with Vivek Krishnan, Sher Ali, Albert Gonzales and Pratish Thakore as joint first authors – report that one member of the STIM family, STIM1, plays an important role in arterial smooth muscle cells that is different from the canonical STIM pathway (Krishnan et al., 2022).

Krishnan et al. first genetically modified mice so that they would not produce STIM1 in their smooth muscle cells. They then used state-of-the-art microscopy techniques, which can image cellular structures only a few nanometers in size, to establish that the arterial smooth muscle cells of these mice had fewer peripheral coupling sites. Loss of STIM1 also altered how some ion channels in the plasma membrane and the SR/ER membrane were clustered, and reduced the ability of calcium released from the SR/ER to activate ion channels in the plasma membrane. These changes made arterial smooth muscle cells less able to contract, which meant that the mice had relaxed arteries and lower blood pressure. These results demonstrate how these microenvironments within arterial smooth muscle cells have global implications. Specifically, the findings suggest that STIM1 may be regulating blood pressure by helping to maintain peripheral coupling sites in arterial smooth muscle cells.

One open question is how this new role for STIM1 compares to its more established canonical signaling pathway. This is especially important given that when arteries become injured, arterial smooth muscle cells shift into a non-contractile, proliferative phenotype to help repair the damage. In this state, these cells use canonical STIM1-dependent SOCE, which is also upregulated during hypertension (Potier et al., 2009; Zhang et al., 2011; Johnson et al., 2020). This implies that arterial smooth muscle cells are capable of using STIM1 in its canonical and non-canonical roles depending on their state (Figure 1).

Further research will be needed to determine exactly how the absence of STIM1 leads to the loss of peripheral coupling sites. One explanation is that STIM1 normally acts as a bridge between the SR/ER and plasma membranes, helping to maintain these tiny regions. Another could be that STIM1 interacts with proteins other than Orai in the plasma membrane, as shown in other cell types (Berlansky et al., 2021). Such interactions may not only form peripheral coupling sites, but also stabilize clusters of these membrane proteins.

Several other questions remain. First, experimental limitations meant it was not possible to directly measure the properties of STIM1 clusters at peripheral coupling sites: doing so could shed light on how these regions form. Second, removing STIM1 from arterial smooth muscle cells reduced the volume of the SR/ER in these cells, and the mechanisms that govern this change are uncertain. Earlier work from some of the scientists involved in the Krishnan et al. study suggested that a protein called junctophilin-2 also maintains peripheral coupling sites (Pritchard et al., 2019). It would be interesting to determine if STIM1 and junctophilin-2 support the same peripheral coupling sites or act at different regions, and whether they interact with different proteins.

In conclusion, Krishnan et al. provide fascinating observations into the non-canonical roles of STIM1 in arterial smooth muscle cells, and how this modulates the ability of arteries to contract, relax and regulate blood pressure. As with all great research, these observations raise as many questions as they answer, making this an exciting area to follow – stay tuned!
